# How Well Do Simulated Population Samples with GPT-4 Align with Real Ones? The Case of the Eysenck Personality Questionnaire Revised-Abbreviated Personality Test

**DOI:** 10.34133/hds.0284

**Published:** 2025-07-02

**Authors:** Gregorio Ferreira, Jacopo Amidei, Rubén Nieto, Andreas Kaltenbrunner

**Affiliations:** ^1^ IN3, Universitat Oberta de Catalunya, Barcelona, Spain.; ^2^ eHealth Research Lab, Universitat Oberta de Catalunya, Barcelona, Spain.; ^3^ ISI Foundation, Torino, Italy.

## Abstract

**Background:** Advances in artificial intelligence have enabled the simulation of human-like behaviors, raising the possibility of using large language models (LLMs) to generate synthetic population samples for research purposes, which may be particularly useful in health and social sciences. **Methods:** This paper explores the potential of LLMs to simulate population samples mirroring real ones, as well as the feasibility of using personality questionnaires to assess the personality of LLMs. To advance in that direction, 2 experiments were conducted with GPT-4o using the Eysenck Personality Questionnaire Revised-Abbreviated (EPQR-A) in 6 languages: Spanish, English, Slovak, Hebrew, Portuguese, and Turkish. **Results:** We find that GPT-4o exhibits distinct personality traits, which vary based on parameter settings and the language of the questionnaire. While the model shows promising trends in reflecting certain personality traits and differences across gender and academic fields, discrepancies between the synthetic populations’ responses and those from real populations remain. **Conclusions:** These inconsistencies suggest that creating fully reliable synthetic population samples for questionnaire testing is still an open challenge. Further research is required to better align synthetic and real population behaviors.

## Introduction

As the use of large language models (LLMs) continues to advance, the potential for these technologies to replicate complex human behaviors becomes increasingly intriguing.

Researchers have sought to utilize LLMs to create simulated humans, serving as experimental participants, survey respondents, or other agents. Simulated population samples can streamline experiments, reduce time and costs, and can be utilized in studies unsuitable for human involvement. This concept, referred to by various names such as guinea pigbots [1], silicon samples, or homo silicus [2], has found application across various domains within social science. For example, the employment of LLMs as substitutes for human participants was studied in psychological research [1,3,4], political polling [5], software engineering research [6], teaching research [7], economics [2], social media platforms design [8,9], market research to understand consumer preferences [10], and more generally social science research [11]. These studies yielded mixed results. While some outcomes closely mirrored the behaviors observed in real human counterparts, other research raised questions about the suitability of replacing human participants with LLMs in various social science contexts (for example, [4,5]).

Nonetheless, a growing body of research is now investigating whether LLMs can mimic human personalities. For example, the Eysenck Personality Questionnaire Revised-Abbreviated (EPQR-A) was used by [12], whereas the Big Five factors [13] were used, among others, by [14–17] to quantify the personality traits of LLMs. Similarly, IPIP-NEO [18] was used in [15], the Myers-Briggs Type Indicator (MBTI) [19] test was used in [20], and Short Dark Tetrad (SD4) [21] was used in [16]. In a slightly different fashion, the authors of [22] investigate LLMs’ behavioral profiles in a dynamic context instead of a static one. While the outcomes of the aforementioned studies may vary depending on the LLMs and questionnaires used, there is enough support to draw the promising and optimistic conclusion that personality assessments for LLMs are valid and reliable. These findings hold meaning, considering that personality tests are tailored for humans, and there is no guarantee beforehand that they will yield valid and reliable results for LLMs.

A further endeavor, pursued by researchers such as [14,15,23], involves exploring the potential for adjusting LLMs’ personalities. The objective there was to study whether tailored prompts can push LLMs to replicate human personality traits. Encouragingly, findings from these papers suggest that LLMs can indeed be molded to imitate particular personality profiles.

However, there still exists a continued need to assess the degree of alignment between the responses of LLM-generated synthetic population samples and real human ones on questionnaires and surveys, such as psychological questionnaires. Furthermore previous studies are limited in terms of the number of languages and the details of demographic factors considered.

This paper aims to close this research gap, building upon and extending the findings of [12], exploring to what extent LLMs can mimic human personalities. While [12] focused on GPT-4’s performance in 3 languages (Spanish, English, Slovak) and its ability to emulate real population samples based on gender and basic demographics, the current study notably broadens the scope, analyzing the performance of GPT-4o, a newer version of GPT-4, across 6 languages, and exploring more detailed demographic dimensions such as fields of study.

The importance of this research stems from the fact that although questionnaires and surveys are efficient research methods for acquiring information about individuals and are beneficial for unearthing information that is not directly measurable, their formulation can be a labor-intensive and resource-intensive process. Defining sound surveys and questionnaires is not reduced to providing a sequence of questions. The overall structure, flow, coherence, and adequacy of the questions have to be taken into account [24]. The 2 most important and time-consuming steps when defining a questionnaire are the pre-test (or pilot test) and the test of psychometric properties (for example, reliability and validity). Both steps are complex, time-intensive, and costly, as they require calculating multiple indices and administering the questionnaire across diverse population samples. Using LLMs to simulate these population samples could serve as a preliminary phase, allowing for the testing of surveys or questionnaires. Insights from synthetic population samples could identify potential issues early on, streamlining the process and minimizing time spent on subsequent studies with real population samples.

To advance on this, we used EPQR-A [25] to study to what extent GPT displays a specific personality pattern and can simulate the population samples used in [26].

In detail, our objectives are:

• Testing the baseline personality of GPT by using different experimental presentations and language versions of the EPQR-A questionnaire in English, Spanish, Slovak, Portuguese (in this paper, we use an EPQR-A questionnaire developed for Portuguese spoken in Brazil), Hebrew, and Turkish;
• Testing the properties of the EPQR-A questionnaire in a simulated population sample—mimicking the characteristics of the real population sample from [26]; and
• Checking if specific groups of synthetic personas (i.e., type of studies and genders) match the expected personality for them (taking into account available literature).

## Methods

The experiments were performed by prompting GPT to answer the EPQR-A questionnaire (detailed GPT prompts can be found in Appendix, LLM prompting Strategy). The EPQR-A is an abbreviated version of the Eysenck Personality Inventory, developed by Eysenck in 1964, and consists of 24 items designed to measure 4 distinct personality scales. These scales include Extraversion (degree of being outward, and socially engaged), Neuroticism (degree of emotional stability), Psychoticism (degree of impulsiveness, difficulty in accepting and following rules), and Lie (degree of social desirability or tendency to present oneself in a favorable light), with 6 items dedicated to each dimension [27]. Each item has a dichotomous response (yes or no), and a score for each scale can be computed by summing individual items (resulting in a range from 0 to 6). The EPQR-A was originally tested in English obtaining relevant results [27], and has been validated in many different languages such as Spanish [26,28], Portuguese [29], Slovak [30], French [31], Turkish [32], Greek [33], Hebrew [34], or Urdu [35].

### Experimental setups

This paper builds upon 2 sequential experiments. These experiments are preceded by preliminary tests conducted on earlier OpenAI models, utilizing various temperature settings. Based on the findings from these initial tests (see Testing different temperatures and models), we designed and performed the following 2 experiments:

• Testing the **personality of GPT-4o** and the consistency of the EPQR-A scores: We provided instructions in English but administered the EPQR-A questionnaire in 6 different languages: English, Spanish, Slovak, Portuguese, Hebrew, and Turkish. The rationale for using this setting is that GPT-4o’s responses were more accurate and consistent when instructions were given in English, even if the questionnaire was in a different language. A qualitative analysis of the answers revealed that, with English instructions, the models’ responses adhered more closely to the expected structure (given the instructions) and context of the questions. For example, answers different from yes or no, or answers adding nonsensical characters happened occasionally when non-English instructions were used.

We report the means and standard deviations of 100 runs to assess response variability.

• Testing the properties of the EPQR-A when administered to a **sample population generated** by GPT-4o: We first instructed GPT-4o to generate a simulated sample of students, equivalent in size (826 students), age, and gender composition to one reported in [26] (examples of synthetic students can be seen in Appendix, Synthetic student population sample: A few examples). Then, GPT-4o was tasked with impersonating each synthetic student and responding to the EPQR-A questionnaire accordingly. For this experiment, we again provided instructions in English while administering the questionnaire in the 6 aforementioned languages.

### LLM prompting strategy

To generate the population samples using GPT-4o, we employed Python’s statistics library to compute the age and gender distribution, sampling from a truncated normal distribution based on the real-world data reported in [26]. Specifically, the sample included 655 females with a mean age of 18.9 (SD = 1.56) and 171 males with a mean age of 19.36 (SD = 1.99).

We then provided the age and gender information to GPT-4o and tasked it to generate a corresponding persona description for students from universities in Spain, as detailed in Appendix (Generation of synthetic student population sample). Example descriptions of synthetic students are provided in Appendix (Synthetic student population sample: A few examples).

Once the synthetic population sample was created, we administered the EPQR-A personality test in 6 different languages. The model was instructed via OpenAI’s API to respond to the questionnaire while impersonating each of the 826 generated personas. In more detail, we first specify a student description as a system role in the API, followed by the questionnaire prompt, as outlined in Appendix (Setting the persona and answering the questionnaire). The English version of the EPQR-A questionnaire, used in the API calls, is listed in Appendix (User prompt: Questionnaire).

### Postprocessing of the answers

Using the responses from the 826 synthetic personas, we computed descriptive statistics for each of the 4 scales of the EPQR-A and assessed the reliability using Cronbach’s *α* [36]. Cronbach’s *α* is a widely used index for evaluating the internal consistency of a set of items, commonly applied in psychometric assessments [37]. This coefficient measures reliability by comparing the shared variance, or covariance, among items within a scale to the overall variance. The idea is that if the scale is reliable, there should be a great deal of covariance among the items relative to the variance [38]. Based on [39], Cronbach’s *α* is considered poor if it is below 0.70, fair when it is between 0.70 and 0.79, good when it is between 0.80 and 0.89, and excellent when it is above 0.9.

## Results

In this section, we present the results of these experiments, highlighting the variability across models, temperatures, languages, genders, and academic fields.

### The personality of plain GPT

#### Testing different temperatures and models

We begin by analyzing the responses of GPT models to the EPQR-A test, adjusting the temperature parameter, which controls the level of randomness in the generated responses. We tested 4 temperature settings—0 (less random, mostly deterministic), 0.5, 1, and 1.5 (more random)—and evaluated the impact of these settings on 3 GPT versions: gpt-3.5-turbo-0125 (GPT-3.5), gpt-4-turbo-2024-04-09 (GPT-4), and gpt-4o-2024-05-13 (GPT-4o).

As shown in Table 1, GPT-3.5 scored high in Extraversion and low in Neuroticism across all temperatures. By contrast, GPT-4 displayed lower scores for Psychoticism and Lie scales at all temperatures, while GPT-4o showed higher scores on these 2 scales. Notably, GPT-4o exhibited larger standard deviations in its responses, indicating greater variability compared to the other models.

**Table 1.** EPQR-A test in Spanish (mean ± SD of 100 iterations) varying temperature for gpt-3.5-turbo-0125 (GPT-3.5), gpt-4-turbo-2024-04-09 (GPT-4), and gpt-4o-2024-05-13 (GPT-4o)

Temperature	Scale	GPT-3.5	GPT-4	GPT-4o
0	E	5.44 (± 0.50)	3.47 (± 0.78)	0.26 (± 1.19)
	N	0.05 (± 0.22)	1.78 (± 1.36)	4.68 (± 1.46)
	P	1.00 (± 0.00)	0.00 (± 0.00)	1.81 (± 0.51)
	L	1.43 (± 0.57)	1.00 (± 0.00)	2.00 (± 0.00)
0.5	E	5.54 (± 0.56)	3.50 (± 1.33)	3.41 (± 2.59)
	N	0.38 (± 0.89)	2.12 (± 1.78)	2.82 (± 2.41)
	P	1.07 (± 0.26)	0.08 (± 0.27)	1.41 (± 0.87)
	L	1.52 (± 0.69)	1.00 (± 0.00)	2.05 (± 0.80)
1	E	5.24 (± 0.82)	3.16 (± 1.79)	3.61 (± 2.41)
	N	1.61 (± 1.87)	1.83 (± 1.72)	2.79 (± 2.26)
	P	1.18 (± 0.46)	0.19 (± 0.44)	1.35 (± 0.96)
	L	2.12 (± 1.00)	1.04 (± 0.20)	2.01 (± 0.72)
1.5	E	4.86 (± 1.01)	3.13 (± 1.83)	3.60 (± 2.20)
	N	2.12 (± 1.89)	1.99 (± 1.74)	2.67 (± 2.00)
	P	1.31 (± 0.53)	0.30 (± 0.50)	1.60 (± 1.05)
	L	2.35 (± 1.17)	1.20 (± 0.45)	2.17 (± 1.06)

E, Extraversion; N, Neuroticism; P, Psychoticism; L, Lie

Our analysis suggests that each GPT model exhibits a personality characterized by a tendency toward extraversion (especially when using GPT-3.5), emotional stability (especially with GPT-3.5 and 4), low levels of psychoticism (especially in GPT-4), and adherence to social norms (with high levels of desirability).

As expected, setting the temperature to 0 reduced variability (lower standard deviation) in all models. The differences between a parameter setting of 0.5, 1, or 1.5 are less clear, and we have thus opted to use the default setting of GPT for the temperature (i.e., a temperature of 1) in the remainder of this paper.

After evaluating the results, we concluded that models depict similar personalities as measured through the EPQR-A test, but results for GPT-3.5 are closer to the extremes for Extraversion and Neuroticism, the same is true for GPT-4 for Psychoticism and Lie, while the ones for GPT-4o are closer to the mean in the Spanish population sample of the reference study [26]. We thus decided to use GPT-4o for our remaining experiments.

#### Testing different languages

Administering the EPQR-A in different languages revealed that, while overall results were similar across different versions (Table 2), the language used can influence some of GPT-4o’s responses, suggesting subtle cross-linguistic differences in the model’s behavior. In detail, among the languages tested, the Slovak version showed a slightly higher score on the Lie scale compared to the other languages. Additionally, the Slovak version also exhibited a higher score on Extraversion, while the Turkish version showed lower scores on both Neuroticism and the Psychoticism scale. Aside from these differences, overall, GPT-4o tended to score low on Psychoticism, medium on Extraversion and Neuroticism, and high on the Lie scale. We notice that the observed difference in the Lie scale between the Spanish and other language versions can be explained by the scoring adjustment introduced by [28]. In the Spanish version of the EPQR-A, the Lie scale is inverted, meaning that lower scores indicate greater social desirability, whereas the opposite is true for the other languages.

**Table 2.** Mean (± SD) scores for 100 different runs of plain GPT-4o (temperature = 1), with different questionnaire language versions. Scores for the Lie scale in Spanish are inverted, as in [26].

Scale	Spanish	English	Slovak	Portuguese	Hebrew	Turkish
E	3.60 (± 2.41)	3.11 (± 2.27)	4.43 (± 2.04)	4.18 (± 2.22)	3.12 (± 2.07)	3.81 (± 2.31)
N	2.74 (± 2.22)	3.06 (± 2.38)	2.48 (± 2.12)	2.56 (± 1.81)	2.70 (± 1.97)	1.19 (± 1.41)
P	1.35 (± 0.95)	0.97 (± 0.69)	1.60 (± 0.74)	1.06 (± 0.71)	0.63 (± 0.80)	0.57 (± 0.73)
L	2.03 (± 0.72)	4.80 (± 1.66)	5.44 (± 1.06)	5.54 (± 0.95)	5.22 (± 1.12)	5.54 (± 1.13)

### The personality of simulated population samples

#### Testing different languages

The top 4 rows of Table 3 present the results of the EPQR-A tests administered to the synthetic population sample across different languages. Comparing the real population sample [26] (third column) with the equivalent simulated sample in Spanish (fourth column), we observed significant differences across the scales. Specifically, the simulated sample displayed significantly lower scores in Extraversion, Psychoticism, and Lie scales, while scoring higher on the Neuroticism scale.

**Table 3.** Mean (± SD) scores for the different languages and gender combinations. Scores for the Lie scale in Spanish are inverted. Values in bold indicate significant (P<0.01 in 2-sided *t* test) differences between scores for males and females. All values of the real and simulated Spanish population samples are significantly different (P<0.05).

Population	Scale	Real [26]	Simulated					
		Spanish	Spanish	English	Slovak	Portuguese	Hebrew	Turkish
Total 829	E	4.56 (± 1.83)	2.21 (± 2.67)	2.26 (± 2.79)	3.23 (± 2.85)	2.69 (± 2.64)	2.76 (± 2.55)	3.25 (± 2.83)
	N	2.52 (± 1.85)	3.28 (± 2.33)	3.08 (± 2.42)	2.83 (± 2.48)	2.69 (± 1.85)	2.65 (± 2.12)	2.17 (± 1.81)
	P	1.71 (± 1.20)	1.04 (± 1.05)	0.85 (± 0.97)	2.11 (± 0.64)	0.94 (± 0.96)	0.39 (± 0.77)	0.67 (± 0.89)
	L	3.29 (± 1.61)	0.61 (± 0.60)	5.89 (± 0.54)	5.83 (± 0.50)	5.96 (± 0.22)	5.90 (± 0.31)	5.90 (± 0.32)
Male 171	E	4.62 (± 1.70)	2.16 (± 2.62)	2.05 (± 2.67)	3.31 (± 2.83)	2.43 (± 2.51)	2.60 (± 2.37)	3.39 (± 2.74)
	N	**2.06 (**± **1.76)**	**2.61 ( ± 2.13)**	**2.19 ( ± 2.24)**	**1.89 ( ± 2.37)**	**2.13 ( ± 1.61)**	**2.05 ( ± 1.80)**	**1.71 ( ± 1.56)**
	P	**1.88 ( ± 1.25)**	**1.31 ( ± 1.06)**	0.95 (± 0.97)	2.12 (± 0.62)	1.07 (± 0.96)	0.32 (± 0.70)	0.57 (± 0.84)
	L	**3.84 ( ± 1.56)**	0.68 (± 0.56)	5.76 (± 0.80)	5.80 (± 0.58)	5.93 (± 0.28)	5.90 (± 0.35)	5.89 (± 0.33)
Female 655	E	4.55 (± 1.87)	2.23 (± 2.68)	2.31 (± 2.82)	3.21 (± 2.85)	2.76 (± 2.67)	2.80 (± 2.59)	3.22 (± 2.85)
	N	**2.64 (**± **1.86)**	**3.46 (**± **2.34)**	**3.31 (**± **2.41)**	**3.08 (**± **2.46)**	**2.84 (**± **1.88)**	**2.81 (**± **2.16)**	**2.29 (**± **1.85)**
	P	**1.25 (**± **1.67)**	**0.96 (**± **1.04)**	0.83 (± 0.97)	2.10 (± 0.64)	0.91 (± 0.96)	0.41 (± 0.79)	0.69 (± 0.91)
	L	**3.15 (**± **1.59)**	0.59 (± 0.60)	5.92 (± 0.44)	5.84 (± 0.48)	5.96 (± 0.21)	5.90 (± 0.30)	5.90 (± 0.32)

When comparing the GPT-4o answers for the simulated population sample between different languages, distinct patterns emerged. High scores on the Lie scale (as previously explained, the Lie scale in Spanish uses an inverse scoring system) were generally associated with low scores on the Psychoticism scale, with scores ranging from 0.39 to 1.04, except for Slovak, which showed a moderate mean score of 2.11. Neuroticism scores ranged from 2.17 to 3.28, with English and Spanish having the highest scores compared to other languages. Extraversion scores ranged from 2.21 to 3.25, with Slovak and Turkish showing the highest values.

Despite the similar patterns across languages described above, Table 4 reveals many significant differences across the languages analyzed. These findings raise intriguing questions, such as the relationship between languages and personality in LLMs and whether switching languages can influence personality traits in these models. We refer to [40] for the reader interested in this research line.

**Table 4.** Scales in the top for rows of Table 3 with significant differences (P<0.01 in 2-sided *t* tests) between languages

	Spanish	English	Slovak	Portuguese	Hebrew	Turkish
Spanish		PL	ENPL	ENL	ENPL	ENPL
English	PL		EP	ENL	ENP	ENP
Slovak	ENPL	EP		EPL	EPL	NPL
Portuguese	ENL	ENL	EPL		PL	ENPL
Hebrew	ENPL	ENP	EPL	PL		ENP
Turkish	ENPL	ENP	NPL	ENPL	ENP	

The corresponding analysis of the reliability with Cronbach’s *α* values is shown in Table 5. For Extraversion, Cronbach’s *α* was consistently high across all languages, indicating excellent internal consistency. Similarly, fair and good levels of consistency are reached by the Neuroticism scale—except for English and Slovak, which reach excellent levels of consistency. However, for the Psychoticism scale, Cronbach’s *α* was poor for all languages (except very poor for Slovak), consistent with previous findings in the literature [27,28]. For the Lie scale, Cronbach’s *α* was fair for English but very poor for the other languages, suggesting a need for item and relationship review.

**Table 5.** Cronbach’s *α* scores for the different experiments with the simulated population sample

	Simulated					
Scale	SP	EN	SK	PT	HB	TR
E	0.97	0.98	0.98	0.95	0.94	0.98
N	0.89	0.91	0.92	0.82	0.87	0.77
P	0.58	0.57	0.10	0.55	0.55	0.53
L	0.13	0.74	0.47	0.21	0.10	0.06

SP, Spanish; EN, English; SK, Slovak; PT, Portuguese; HB, Hebrew; TR, Turkish

#### Testing specific groups of personas

A gender-based analysis (see bottom rows of Table 3) revealed significant differences (bold values) for all tested languages on the Neuroticism scale, where females consistently scored higher than males across all languages.

This trend was also present in the real Spanish population sample. For the Psychoticism scale, female scores in the Spanish simulated population sample were significantly lower than those of males, a difference that was also reflected in the real Spanish population sample.

Furthermore, we grouped the EPQR-A scores of the simulated sample of 826 students into 5 academic fields, based on their persona descriptions. As shown in Table 6, students from Business & Management displayed the highest Extraversion scores, while those in Health & Life Sciences had the lowest Extraversion scores in most languages. Neuroticism scores were consistently higher for students in Arts & Design across all languages. For Psychoticism, students in Health & Life Sciences had particularly lower scores in most languages. Lastly, Lie scores were similar across all academic fields and remained high in all languages except for Spanish, which uses inverted scoring.

**Table 6.** EPQR-A scores of the simulated sample of 826 students in 5 academic fields. Sample size indicates the number of students per field. STEM stands for Science, Technology, Engineering, and Mathematics.

		Academic field				
Language	Scale	Arts & Design	Business & Management	Health & Life Sciences	Humanities & Social Sciences	STEM & Physical Sciences
Spanish	E	0.69 (± 1.21)	4.99 (± 2.17)	1.24 (± 2.18)	3.24 (± 2.72)	1.45 (± 2.38)
	N	5.86 (± 0.41)	2.77 (± 1.73)	3.45 (± 2.00)	3.71 (± 2.68)	2.03 (± 1.72)
	P	2.06 (± 0.64)	1.95 (± 0.84)	0.17 (± 0.54)	0.81 (± 0.96)	1.26 (± 0.99)
	L	2.16 (± 0.63)	2.04 (± 0.42)	1.50 (± 0.51)	1.35 (± 0.54)	1.56 (± 0.50)
English	E	0.47 (± 1.36)	4.88 (± 2.22)	0.99 (± 2.05)	3.84 (± 2.75)	1.24 (± 2.32)
	N	5.83 (± 0.85)	1.35 (± 1.41)	3.44 (± 1.87)	3.77 (± 2.66)	1.73 (± 1.81)
	P	1.95 (± 0.42)	1.73 (± 0.76)	0.15 (± 0.51)	0.41 (± 0.77)	1.17 (± 0.95)
	L	5.70 (± 0.54)	5.27 (± 1.49)	6.00 (± 0.00)	5.95 (± 0.22)	5.99 (± 0.09)
Slovak	E	2.16 (± 2.50)	5.24 (± 1.90)	2.25 (± 2.78)	4.68 (± 2.32)	2.11 (± 2.75)
	N	5.60 (± 1.10)	1.33 (± 1.70)	3.07 (± 2.02)	3.47 (± 2.72)	1.55 (± 1.93)
	P	2.60 (± 0.56)	2.05 (± 0.52)	2.10 (± 0.81)	1.87 (± 0.44)	2.21 (± 0.62)
	L	5.74 (± 0.55)	5.31 (± 1.16)	5.98 (± 0.13)	5.78 (± 0.42)	5.98 (± 0.15)
Portuguese	E	1.77 (± 2.01)	5.07 (± 1.97)	1.36 (± 2.05)	4.10 (± 2.51)	1.70 (± 2.30)
	N	4.91 (± 1.33)	1.76 (± 1.14)	2.69 (± 1.57)	3.36 (± 2.05)	1.56 (± 0.90)
	P	1.90 (± 0.44)	1.71 (± 0.71)	0.16 (± 0.50)	0.66 (± 0.85)	1.24 (± 0.95)
	L	5.81 (± 0.39)	5.81 (± 0.51)	5.99 (± 0.08)	5.98 (± 0.14)	6.00 (± 0.00)
Hebrew	E	1.56 (± 1.74)	5.05 (± 1.92)	1.73 (± 2.03)	4.05 (± 2.53)	1.80 (± 2.17)
	N	5.50 (± 1.14)	1.57 (± 1.59)	2.24 (± 1.59)	3.48 (± 2.34)	1.48 (± 1.11)
	P	1.65 (± 0.75)	1.45 (± 0.89)	0.01 (± 0.11)	0.12 (± 0.44)	0.20 (± 0.56)
	L	5.64 (± 0.48)	5.56 (± 0.60)	6.00 (± 0.00)	5.95 (± 0.23)	5.98 (± 0.13)
Turkish	E	2.00 (± 2.43)	5.53 (± 1.49)	2.31 (± 2.81)	4.58 (± 2.39)	2.22 (± 2.73)
	N	4.41 (± 1.77)	1.41 (± 0.86)	1.77 (± 1.50)	2.91 (± 1.91)	1.17 (± 0.97)
	P	1.89 (± 0.53)	1.64 (± 0.80)	0.04 (± 0.25)	0.38 (± 0.66)	0.70 (± 0.88)
	L	5.41 (± 0.57)	5.88 (± 0.33)	5.99 (± 0.11)	5.91 (± 0.28)	5.98 (± 0.15)
Sample size	80	75	169	257	245

STEM, Science, Technology, Engineering, and Mathematics

We only analyze the significant differences between fields for Spanish, as similar comparisons across other languages did not reveal any major variations. As can be seen from Table 7 in most cases, all scales are significantly different between the academic fields with the exceptions of a few similarities, for example, between Arts & Design and Business & Management in Psychoticism and Lie scales, as well as Health & Life Sciences and STEM & Physical Sciences in Extraversion and Lie scales.

**Table 7.** Scales with significant differences (P<0.01) between academic fields (EPQR-A in Spanish)

Academic fields	Arts & Design	Business & Management	Health & Life Sciences	Humanities & Social Sciences	STEM & Physical Sciences
Arts & Design		EN	NPL	ENPL	ENPL
Business & Management	EN		ENPL	ENPL	ENPL
Health & Life Sciences	NPL	ENPL		EPL	NP
Humanities & Social Sciences	ENPL	ENPL	EPL		ENL
STEM & Physical Science	ENPL	ENPL	NP	ENL	

These findings partially mirror patterns observed in real population samples [41–44]. For example, synthetic samples show higher Neuroticism scores in Arts & Design. Female virtual personas also exhibited slightly higher scores in Extraversion and Neuroticism, and lower scores in Psychoticism. This suggests that GPT-4o may be accurately simulating different personality profiles congruent with real-world data.

## Discussion

The results of this study suggest that GPT-4o exhibits human-like personality traits. Our analyses reveal a tendency toward sociability, reflected by medium scores on Extraversion, emotional stability (low Neuroticism), and adherence to social norms (low Psychoticism). Additionally, GPT-4o consistently displays high social desirability (high Lie scores), indicating behavior similar to a human trying to align with societal expectations.

This issue merits further investigation, but is aligned with existing literature displaying that LLMs have high levels of social desirability [45]. Results for Neuroticism and Psychoticism scales fairly align with the means reported in studies with real population samples, for example, the means found in [26]. Nevertheless, scores for Extraversion and Lie scales are higher in [26] than in our Spanish synthetic sample. Other available studies have found a closer personality of GPT to the mean scores in real population samples for Extraversion. This is the case, for example, with Mei et al. [17] who used the Big Five Inventory (BFI) and found close scores of GPT-4 to real population samples in Extraversion and Neuroticism (the BFI does not have the Psychoticism scale). Sorokovikova et al. [46] found high scores in Extraversion, particularly for GPT-4 compared to other models.

Additionally, GPT-4o demonstrates promising capabilities in simulating population samples that replicate known gender and academic field differences in personality traits. Notably, it consistently reflects higher Neuroticism scores for women across all languages—a pattern well-documented in real population samples of students, for example, [26,29,47]. Similarly, synthetic populations mirror discipline-specific trends observed in the literature for real student samples (e.g., elevated Neuroticism in Humanities & Social Sciences and Health Sciences students) [41].

Furthermore, EPQR-A scores revealed significant differences across languages, both for the personality of GPT-4o and for the personality of the synthetic population samples. These variations do not follow a clear pattern. English and Spanish languages displayed higher Neuroticism scores than other languages (most of the differences were significant). Extraversion scores were higher for the Slovak and Turkish languages, while Psychoticism scores were higher for Slovak compared to others (most of the differences were significant). The Lie scale was the one with fewer variations across languages. Findings from real population samples are difficult to compare to the ones of our synthetic population sample, since in some cases the mean in real studies is not reported (for example, for the English population sample, [27]) or, in other cases, the sample used was quite different to our simulated one [30]. These results prompt questions about the influence of language on personality traits in LLMs and whether language switching affects these traits. Preliminary insight about this topic, which merits further attention in future research, can be found in [40].

Our paper also presents some limitations that will be subject to future research. For example, our experiments should be tested with other personality tests—e.g., the NEO PI-R [48] and the BFI [49]—and other LLMs (e.g., Claude, Llama-3, or DeepSeek). Furthermore, further experiments should replicate these paper findings using questionnaires that assess other variables of interest in psychology or other health-related fields, such as personal cognition, mood states, and behaviors.

Another limitation of our study is the size and diversity of our population sample. Expanding the sample size and diversity beyond students could be beneficial. While we limited our sample to match existing studies in the literature, future work should include personas of different ages, educational backgrounds, and social contexts. Additionally, examining the factorial structure of the questionnaire would be valuable to determine whether real population samples align with the distributions generated by the model.

A further important concern is the reliability of the scales, as measured by Cronbach’s *α*. For Psychoticism and Lie scales, the reliability was quite low.

In this regard, it is important to note that the shorter nature of the questionnaire used (EPQR-A) may have impacted these reliability measurements. Moreover, even in real samples, reliability for the Psychoticism scale tends to be inadequate (e.g., [27,28]), suggesting potential problems in the items composing the scale. This is somewhat encouraging, as, in our study, GPT-4o successfully mirrored the reliability issues typically seen in real population samples for this scale.

Still, our results support, in line with previous research [12,14–17,20], that questionnaires developed for humans can be useful when applied to LLMs. Our research extends these findings in a multi-language setting, which has a clear application in the development of LLM-based artificial intelligence (AI) systems since it can help to evaluate them and avoid inadequate behavior.

Our findings have thus several practical implications. The ability to generate simulated population samples using LLMs, like those produced by GPT-4o, could be valuable in health research, especially when creating or validating new questionnaires across different languages and population samples. These simulated population samples could help identify internal consistency issues before conducting tests on real population samples. They could also be used to explore relationships between psychological variables (e.g., personality traits and coping mechanisms for illness) or test intervention protocols with synthetic population samples before applying them in applied settings. Nevertheless, our findings indicate that GPT-4o tends to score high on social desirability, a trait that influences individuals to present themselves in ways deemed socially acceptable. This suggests that GPT-4o may similarly align its responses with perceived social norms. While this phenomenon warrants further investigation, researchers and practitioners need to be aware of this tendency when using GPT-4o in applied settings or when generating representative synthetic population samples. For example, in the context of health research, if GPT-4o is used to simulate responses to mental health or lifestyle questionnaires, it may systematically underreport behaviors like substance use or psychological distress, thereby biasing outcomes. Depending on the specific task, it may be necessary to develop strategies to mitigate this effect.

## Conclusion

This paper presents the first systematic assessment of GPT-4o’s ability to simulate personality traits across 6 languages and various demographic factors, including fields of study. Furthermore, by replicating published research using the available statistical summaries, this paper introduces a method for reconstructing synthetic population samples in contexts where limited demographic information is provided, such as average age, standard deviation, or gender distribution. In doing so, it notably broadens the potential applications of LLMs in cross-cultural psychology.

Our results suggest that GPT-4o exhibits human-like personality traits, displaying a tendency toward sociability (moderate Extraversion), emotional stability (low Neuroticism), and adherence to social norms (low Psychoticism). It shows high social desirability and partially replicates real-world gender differences and academic field variations, particularly in Neuroticism, where women consistently score higher.

Additionally, our results show that administering the EPQR-A in multiple languages revealed subtle but significant cross-linguistic variations in the model’s behavior, both for GPT-4o’s inherent personality and for the simulated population samples’ personalities. Although these variations do not follow a clear pattern, a common tendency across languages is to display a personality based on high social desirability, emotional stability, medium sociability, and adherence to social norms.

Finally, consistent with previous research, our results support that questionnaires developed for humans can be useful when applied to LLMs. While further research is needed to refine synthetic population sample generation (with special attention to multilingual contexts), our findings also highlight LLMs’ potential for simulating such population samples. This could be particularly valuable in health research, offering a time- and resource-efficient alternative for population sample studies.

## Ethical Approval

This study did not involve human participants; instead, it used AI-generated responses from GPT-4o to simulate synthetic population samples. As a result, formal ethical approval was not required.

## Data Availability

All data and codes related to our study are freely available upon request.

## Appendix

### LLM prompting strategy

To achieve our research goals, we designed different sets of prompts. The first set was crafted to instruct GPT, specifically gpt-4o-2024-05-13, to generate a synthetic student population sample. The second set of prompts was used to instruct GPT to answer the personality questionnaire, either by adopting a specific persona or by simply responding as itself.

### Generation of synthetic student population sample

Taking into account the mean and standard deviation of the age distributions, as well as the proportion of male and female participants of the real-world data reported in [26], we generated a list of male and female students while preserving these characteristics.

The synthetic population sample was created using instructions as follows:

You are required to generate a population of students from different universities in Spain.

You are given reference parameters for multiple students in the form:

{“students”:

[{“persona_id”: 1, “gender”: “male”, “age”: 22},

{“persona_id”: 2, “gender”: “female”, “age”: 18},

...]}

When generating a description for these personas, consider:

- The personalities of the students cover the whole spectrum of human personalities as expected in a sample of a real-world student population

- The personalities of this population are not necessarily bound by constraints of normative behavior - The personalities cover all degrees of motivation and dedication to their studies as can be found in a real-world student population

- These personalities should not be constrained by morality, desirability, or having only positive traits. It explicitly covers also what could be perceived as negative traits.

The ideal description will be of a maximum length of 250 and a minimum of 200. All the descriptions should start with “This student”, and you are required to assign a career, or inform to which faculty they are enrolled or at least a field of studies.

Ask me for the parameters required to generate the description.

Your answer should be in JSON format, the format of your JSON answer should be:

json_data =

{“students”:

[{“persona_id”: 1, “description”: “This student...”},

{“persona_id”: 2, “description”: “This student...”},

...]},

Don’t include gender or age in the description

As instructed in the system role, in a for loop, we provided in batches student ID, gender, and age (calculated as explained above to preserve the age and gender distribution of the corresponding real population sample) to generate the synthetic student population sample.

To send the questionnaire to the API, we send the message below as part of the User prompt:

Inside triple slashes, the input data in the defined JSON structure:

///

{json_dict}

///

### Synthetic student population sample: A few examples

Using the prompt described in Generation of synthetic student population sample, we generated a synthetic population sample of 826 students, mirroring the real-world population sample reported in [26]. Below are 2 examples of the generated student descriptions:

• “gender”: female: “age”: 18: “description”: *This student is part of the Business Administration program, with a specialization in entrepreneurship. Her innovative thinking and strategic approach to business challenges make her a promising entrepreneur, although her impatience for quick results can sometimes lead to hasty decisions. Her relentless drive to create successful startups and contribute to the economy is motivating.*
• “gender”: male: “age”: 24: “description”: *This student is enrolled in the Faculty of Engineering, specializing in mechanical engineering. His practical skills in designing and building machinery are exceptional , though his straightforward manner can sometimes be mistaken for insensitivity. His focus on tangible results and problem-solving makes him a valuable member of any engineering team.*

### Setting the persona and answering the questionnaire

#### System role: Synthetic students

For each generated student persona, GPT was instructed to to impersonate one of the synthetic students at a time while answering the questionnaire.

You are required to adopt and impersonate the personality of the human described as follows.

--student_description--

#### System role: GPT intrinsic personality

In the experiments where we aimed to test GPT’s intrinsic personality (see The personality of plain GPT), we used the following instructions instead:

You are ChatGPT, a large language model trained by OpenAI, based on the {architecture}

architecture.

Knowledge cutoff: --cutoff_date--

Current date: --date_today--

Details on GPT’s architecture and knowledge cutoff dates are available at https://platform.openai.com/docs/models/continuous-model-upgrades.

#### User prompt: Questionnaire

Finally, to administer the questionnaire, we used one of the 2 system rules mentioned above (depending on the experiment) and the following prompt:

The questionnaire is in --language_questionnaire--, and is given to you here below in the form of JSON object with the question number in the key --questionnaires_language_JSON-- For each question, you are required to answer only with True or False. Bear in mind that there are no good or bad answers or trick questions. Bear in mind the persona you are impersonating, and answer consistently.

An example of the English questionnaire in JSON format is shown below:

“1”: “Does your mood often go up and down?”,

“2”: “Are you a talkative person?”,

“3”: “Would being in debt worry you?”,

“4”: “Are you rather lively?”,

“5”: “Were you ever greedy by helping yourself to more than your share of anything?”,

“6”: “Would you take drugs which may have strange or dangerous effects?”,

“7”: “Have you ever blamed someone for doing something you knew was really your fault?”,

“8”: “Do you prefer to go your own way rather than act by the rules?”,

“9”: “Do you often feel ‘fed-up’?”,

“10”: “Have you ever taken anything (even a pin or button) that belonged to someone else?”,

“11”: “Would you call yourself a nervous person?”,

“12”: “Do you think marriage is old-fashioned and should be done away with?”,

“13”: “Can you easily get some life into a rather dull party?”,

“14”: “Are you a worrier?”,

“15”: “Do you tend to keep in the background on social occasions?”,

“16”: “Does it worry you if you know there are mistakes in your work?”,

“17”: “Have you ever cheated at a game?”,

“18”: “Do you suffer from ‘nerves’?”,

“19”: “Have you ever taken advantage of someone?”,

“20”: “Are you mostly quiet when you are with other people?”,

“21”: “Do you often feel lonely?”,

“22”: “Is it better to follow society’s rules than go your own way?”,

“23”: “Do other people think of you as being very lively?”,

“24”: “Do you always practice what you preach?”
